# Improving Door-to-ECG Time in Acute Myocardial Infarction: A Closed-Loop Audit from a Tertiary Care Center in Peshawar

**DOI:** 10.12669/pjms.41.7.11635

**Published:** 2025-07

**Authors:** Muhammad Ishaq Khan, Abidullah, Muhammad Mujtaba, Nazia Waheed

**Affiliations:** 1 Muhammad Ishaq Khan, Fellow interventional cardiology, Peshawar Institute of Cardiology, Peshawar, Pakistan; 2 Abidullah, Head of Department Cardiology Unit, Peshawar Institute of Cardiology, Peshawar, Pakistan; 3 Muhammad Mujtaba, House Officer, Naseer Teaching Hospital, Peshawar, Pakistan; 4 Nazia Waheed, Resident Cardiology, Peshawar Institute of Cardiology, Peshawar, Pakistan

**Keywords:** Acute myocardial infarction, ECG, Electrocardiogram, STEMI, ST-elevation myocardial infarction, Audit

## Abstract

**Background & Objective:**

Acute myocardial infarction is the leading cause of heart disease worldwide. Timely diagnosis and decision of management plan is paramount which is pinned on early electrocardiogram to minimize the door-to-electrocardiogram time. Our objective was to evaluate the current practice of timing of initial electrocardiogram of patients presenting with Acute myocardial infarction at our local cardiac hospital in Pakistan and implement changes with a complete closed loop audit.

**Methods:**

Retrospective and prospective data collection for all patients who presented to the emergency room of Peshawar Institute of Cardiology from September 2022 till September 2023. The hospital management information system and electronic medical record of the hospital was used for data collection.

**Results:**

A total of 149 patients’ data was collected and analyzed. The first cycle of the audit analyzed 29 patients, the second cycle of the audit analyzed 60 patients and the third cycle of the audit again analyzed 60 patients. In the first set of patients, 17.2 percent of patients had door-to-ECG time of less than 10 minutes which was far from ideal. Therefore different departmental meetings were conducted and it was decided to set up a designated triage for ACS patients. Therefore in the second cycle of the audit 43.3% of patients achieved this time showing improvement. During the third cycle when the initial registration and formal paperwork for admission to ER were removed then 75% of patients had their ECG done within 10 minutes of the presentation.

**Conclusion:**

This audit successfully identified the modifiable factors responsible for the delay in the first ECG which included a lack of staff, non-viability of triage, and paperwork. After working on these factors our target Door to ECG time of 75% was achieved.

## INTRODUCTION

Coronary artery disease remains the leading cause of mortality worldwide^1^, of which acute myocardial infarction (AMI) is a common presentation.^2^ The mainstay of management is early identification and initiating the appropriate protocol in the least time possible.^3^ Leading to the outcome of the patient’s hospital and clinical course, there are different factors^4^ that can be modified to achieve the desired target goal.^5^ One of these is the time delay in obtaining and interpreting the first ECG on a patient’s presentation.

Primary PCI is the first-line treatment strategy for ST-elevation myocardial infarction (STEMI) as recommended by the international guidelines.^6^ It is superior to thrombolysis.^7^ Over the past few decades improvement in care for STEMI patients has been much advocated and is proven to be beneficial after implementing the recommended strategies.^8^ Initiatives for Improvement of care over the past decades have led to the recommendation of performing and interpreting ECG within 10 minutes of arrival for patients suspected to have myocardial ischemia to the emergency department (ED).^6^,^9^ This is shown to have positive effects on reducing door-to-balloon time and hence improving outcomes^10^,^11^ which is the crux of the “*time is muscle*” mantra.

Therefore, even pre-hospital ECG has been vouched for and is encouraged^12^, but our local emergency medical services in the community are not equipped with that facility hitherto, we take the first medical contact FMC at the time of arrival in the ED. We conducted our departmental audit meetings where delays in diagnosis and managing STEMI patients were identified, based on which the demand for establishing an active triage system of identifying the level of urgency and prioritization of patients was raised with the hospital administration. After setting up the triage system we observed significant improvement in door-to-ECG time (D2ET) and also door-to-balloon time (D2BT) complete audit cycles to reduce the D2ET of patients presenting with AMI to the emergency room (ER) of a cardiac hospital and bring it in line with the guidelines recommended practice.

## METHODS

Retrospective and prospective data was reviewed from our local hospital management and information system and electronic medical records of patients presenting to the ER with AMI. Records were searched for the first ECG time and the time of arrival in the ER from 16th September 2022 to 30^th^ September 2023. This audit contains three cycles. The first cycle was from September till 31^st^ December 2023, the 2^nd^ cycle of our audit started from 1^st^ January 2023 till 31^st^ May 2023 the third cycle was started from 1^st^ June 2023 till 30^th^ September. We included only those patients who directly presented to our ER without having had a prior ECG after the onset of symptoms. The standard recommendations identified by the American Heart Association and European Society of Cardiology for D2ET of less than 10 minutes were set as the goal.

Departmental meetings were regularly conducted in order to identify factors in every session and the staff and doctors were educated about the importance and possibility of improvement of D2ET. The meetings decided to appraise the hospital administration of the need for setting up a triage with the required staff and equipment. This was agreed by the hospital administration and a designated triage was started along with trained ECG staff and equipment. Re-audit was performed from 1^st^ January 2023 to 31^st^ May 2023 D2ET of 60 consecutive patients whose record was retrievable. Assessment of the outcomes after implementation of the said intervention were discussed and further improvement was suggested by omitting the paperwork of registration for patients with AMI presenting to the ER. After our 2^nd^ cycle new protocol was formed in which the patients with suspected ACS were excluded from registration paperwork . A third cycle was conducted from June 2023 to September 2023 comprising 60 patients. Statistical software SPSS 20.0 was used for data analysis.

## RESULTS

A total of 149 patients were included. Data is shown in Table I for all the patients. During the first cycle, 17.2% percent of patients had the target D2ET of less than 10 minutes achieved. During the second cycle, the analysis showed that 43.3% of patients had achieved the recommended goal of D2ET. After omitting the paperwork of initial registration during the third cycle, patients’ data was analyzed and it was observed that 75% of patients presenting with AMI to the ER had their ECG done within 10 minutes. The mean time for ECG in the first cycle was 40.68±52.5 mint. The second cycle mean was 12.83±7.97 mint and the third cycle was 9.25±1.80 mint.

**Table I:** Outcomes achieved over each study cycle.

Cycle	<10 mint	>10mint
1^st^ cycle	17.2%	82.8%
2^nd^ cycle	43.3%	56.7%
3^rd^ cycle	75%	25%

## DISCUSSION

In this audit, we found the modifiable factors that were the leading cause of delay in ECG were lack of triage with dedicated staff and equipment. The other factor, which was one of the leading factors, was the registration of patients and entry of the patients into the ED. Living in third-world countries in which we lack the proper channel of referring patients from BHU to tertiary and specialized hospitals, the majority of our patients directly presented to the tertiary care hospital, due to which the care of the patient has been much more compromised. Another point that was noted is the lack of availability of beds in the ED of our government hospital in district Peshawar, which puts a huge burden on the government hospital, as many of the patients presented to the ED of the government hospital cannot afford private hospital charges. To overcome these we first started the initiative after our first cycle in the form of a designated triage and hiring trained staff for ECG, after 2^nd^ cycle we worked on making a paperless entry to the ER so that patients with acute chest pain could directly enter the triage without waiting in que for an ER slip.

Minimizing the time from onset of symptoms and treatment as well as from the FMC to treatment for patients with acute coronary syndrome and their effect on positive outcomes,^11^,^13^ cannot be emphasized more in the current era. The recent guidelines recommend the first ECG to be obtained within 10 minutes of the first medical contact.^6^,^9^ During our audit, we identified different factors that contributed to the delay in diagnosis and management of patients with ACS, especially STEMIs. Controlling these factors could expedite their management. We started by reducing the time to obtain the initial ECG. In the first leg of our audit, we identified that only 17.2% of our patients had their D2ET of less than 10 minutes which was far from ideal. This ultimately leads to the reduction of door-to-balloon time. Furthermore, in patients with cardiogenic shock, 10-minute delay in door-to-balloon time is of more significance than in hemodynamically stable patients.^14^

In ER, patients were inducted directly into the observation where the initial ECG was obtained. After discussion in our departmental meeting, it was decided to set up a dedicated triage where the initial assessment including the first ECG will be obtained. After implementing this we observed a marked reduction in our door to ECG time. The target D2ET was achieved for 75% of patients. The importance of an active^5^ triage in reducing the door-to-ECG and door-to-balloon time has been demonstrated in the literature.^5^,^15^ Improvement of our D2ET would logically improve the total ischemic time and outcomes of our patients.

In March 2012 a clinical audit was carried out about the time for treatment in a suspected case of acute coronary syndrome patients by Khursheed M et al.^16^. According to this clinical audit, the treatment received within 10 minutes patients percentage was 32.9% who presented with acute coronary syndrome. In 2022 another clinical audit was done with the same objective, this audit was conducted in the Armed Force Institute of Cardiology in collaboration with the National Institute of Heart Disease by Sami A et al.^17^, in this audit they identified two major factors that are non-availability of beds in the ER and not attended by doctors at time of presentation. This audit contains only one cycle and in their first cycle, the door to ECG time of a patient who presented with a suspected case of coronary syndrome was 59.4%. As per our literature review, we found out that it is the first of its kind audit that is being held in our region, Peshawar.

Our ECG machines like all other equipment in the hospital are periodically maintained by the biomedical / engineering department which makes tracing of the actual ECG time easier. Moreover, a robust electronic medical record system was vital to collecting data retrospectively. Similarly other factors included awareness of ED staff about the importance of D2E time less than 10 minutes. Our earlier internal audit identified the opportunity to intervene at the very early step of patient presentation, hence the need for a round-the-clock triage system in the ED was emphasized and the effects were dramatic after the implementation.

### Recommendation:

We recommend that the delay in managing patients with AMI can further be reduced by introducing ECG done by trained EMS staff equipped appropriately, at the point of first contact in the community and communicating it to the nearest designated medical facility for diagnosis and initiating the management immediately before arrival of the patient in the medical facility.

### Limitations:

We considered the arrival time in the ER as the first medical contact because we do not have an appropriate ambulance service/emergency medical service in our community. This is a limitation of our project that we could not circumvent.

## CONCLUSION

The first audit cycle successfully identified the modifiable factor in time delay in the first ECG of patients with acute myocardial infarction and its implementation led to improvement in the second cycle. The third cycle showed achievement of 75% compliance in achieving the target door-to-ECG time. This will ultimately lead to improved outcomes by reducing the total ischemia time.

## References

[1] Mensah GA, Fuster V, Murray CJL, Roth GA, Global Burden of Cardiovascular Diseases and Risks Collaborators (2023). Global Burden of Cardiovascular Diseases and Risks, 1990-2022. J Am Coll Cardiol.

[2] Siudak Z, Slawska A, Dziewierz A, Dudek D (2018). P4617 Acute myocardial infarction as a first time presentation of coronary artery disease in Poland in 2014-2016. Eur Heart J.

[3] Byrne RA, Rossello X, Coughlan JJ, Barbato E, Berry C, Chieffo A, ESC Scientific Document Group (2023). 2023 ESC Guidelines for the management of acute coronary syndromes. Eur Heart J.

[4] Feng L, Li M, Xie W, Zhang A, Lei L, Li X (2019). Prehospital and in-hospital delays to care and associated factors in patients with STEMI:an observational study in 101 non-PCI hospitals in China. BMJ Open.

[5] Coyne CJ, Testa N, Desai S, Lagrone J, Chang R, Zheng L (2015). Improving door-to-balloon time by decreasing door-to-ECG time for walk-in STEMI patients. West J Emerg Med.

[6] Members WC, Lawton JS, Tamis-Holland JE, Bangalore S, Bates ER, Beckie TM (2022). 2021 ACC/AHA/SCAI guideline for coronary artery revascularization:a report of the American College of Cardiology/American Heart Association Joint Committee on Clinical Practice Guidelines. J Am Coll Cardiol.

[7] Keeley EC, Boura JA, Grines CL (2003). Primary angioplasty versus intravenous thrombolytic therapy for acute myocardial infarction:a quantitative review of 23 randomised trials. Lancet.

[8] Bradley EH, Nallamothu BK, Herrin J, Ting HH, Stern AF, Nembhard IM (2009). National efforts to improve door-to-balloon time results from the Door-to-Balloon Alliance. J Am Coll Cardiol.

[9] Ibanez B, James S, Agewall S, Antunes MJ, Bucciarelli-Ducci C, Bueno H, ESC Scientific Document Group (2018). 2017 ESC Guidelines for the management of acute myocardial infarction in patients presenting with ST-segment elevation:The Task Force for the management of acute myocardial infarction in patients presenting with ST-segment elevation of the European Society of Cardiology (ESC). Eur Heart J.

[10] Diercks DB, Peacock WF, Hiestand BC, Chen AY, Pollack CV, Kirk JD (2006). Frequency and consequences of recording an electrocardiogram >10 minutes after arrival in an emergency room in non-ST-segment elevation acute coronary syndromes (from the CRUSADE Initiative). Am J Cardiol.

[11] Park J, Choi KH, Lee JM, Kim HK, Hwang D, Rhee TM (2019). KAMIR-NIH (Korea Acute Myocardial Infarction Registry–National Institutes of Health) Investigators. Prognostic Implications of Door-to-Balloon Time and Onset-to-Door Time on Mortality in Patients With ST -Segment-Elevation Myocardial Infarction Treated With Primary Percutaneous Coronary Intervention. J Am Heart Assoc.

[12] Rokos IC, French WJ, Koenig WJ, Stratton SJ, Nighswonger B, Strunk B (2009). Integration of pre-hospital electrocardiograms and ST-elevation myocardial infarction receiving center (SRC) networks:impact on door-to-balloon times across 10 independent regions. JACC Cardiovasc Interv.

[13] Prasad A, Gersh BJ, Mehran R, Brodie BR, Brener SJ, Dizon JM (2015). Effect of ischemia duration and door-to-balloon time on myocardial perfusion in ST-segment elevation myocardial infarction:an analysis from HORIZONS-AMI Trial (Harmonizing Outcomes with Revascularization and Stents in Acute Myocardial Infarction). JACC Cardiovasc Interv.

[14] Scholz KH, Maier SK, Maier LS, Lengenfelder B, Jacobshagen C, Jung J (2018). Impact of treatment delay on mortality in ST-segment elevation myocardial infarction (STEMI) patients presenting with and without haemodynamic instability:results from the German prospective, multicentre FITT-STEMI trial. Eur Heart J.

[15] Su H-Y, Tsai J-L, Hsu Y-C, Lee K-H, Chang C-S, Sun C-K (2021). A modified cardiac triage strategy reduces door to ECG time in patients with ST elevation myocardial infarction. Sci Rep.

[16] Khursheed M, Fayyaz J, Feroze A, Shakeel N, Bhatti JA (2015). Time to treatment in patients of suspected acute coronary syndrome in Pakistan:A clinical audit. Heart Lung.

[17] Sami A, Maqsood S, Iqbal U, Safdar H, Khalil H, Yasir SB (2022). Recording the Door-To-ECG Time for Patients Presenting with Acute Chest Pain to a Cardiac Emergency Unit:A Clinical Audit and Quality Improvement Project. Pak Armed Forces Med J.

